# Open-source compact time-domain hydrogen (1H) NMR System for Field Deployment

**DOI:** 10.1016/j.ohx.2025.e00651

**Published:** 2025-04-29

**Authors:** Winford Janvrin, Jacob Martin, Daniel Hancock, Angelo Varillas, Austin R.J. Downey, Perry J. Pellechia, Joud Satme, Sang Hee Won

**Affiliations:** a University of South Carolina, Department of Mechanical Engineering, Columbia SC, United States; b University of South Carolina, Department of Physics and Astronomy, Columbia SC, United States; c University of South Carolina, Department of Civil Engineering, Columbia SC, United States; d University of South Carolina, Department of Chemistry and Biochemistry, Columbia SC, United States

**Keywords:** Compact Nuclear Magnetic Resonance (NMR), Time-domain NMR (TD-NMR), CPMG, Environmental monitoring, Pulsed NMR system, Hydrogen NMR

## Abstract

This paper presents a compact, low-cost time-domain nuclear magnetic resonance (TD-NMR) system based on a 0.5 T permanent magnet designed for in-situ ${}^{1}$H measurements. Unlike conventional high-field nuclear magnetic resonance (NMR) spectrometers, this system emphasizes relaxation times rather than chemical shifts, enabling material property analysis without large magnets or complex spectral processing. The hardware employs an off-the-shelf data acquisition and control system along with a custom PCB for signal conditioning, ensuring straightforward deployment and reduced costs. The system’s core sequence is a Carr-Purcell-Meiboom-Gill pulse train, chosen for efficient $T_{2}$ relaxation measurements under varying magnetic susceptibilities. By focusing on relaxation measurements, this approach bypasses complexities of high-resolution spectroscopy, enhances signal-to-noise in low-field conditions, and enables robust characterization across challenging environmental settings. We validate the system using aqueous Copper(II) sulfate solutions, correlating T${}_{2}$ values with copper concentrations to simulate environmental heavy metal contamination monitoring. Prior work has demonstrated versatility in fuel property analysis and environmental sensing, confirming broad applicability for this portable platform. While packaging and integration with ancillary equipment (e.g., flow-through systems) are not covered, the platform serves as a versatile foundation for specialized deployments. Its open-source design and affordability aim to democratize NMR technology and extending its utility beyond conventional laboratory environments. This accessible configuration fosters widespread educational and professional use.

## Graphical abstract

**Figure:**
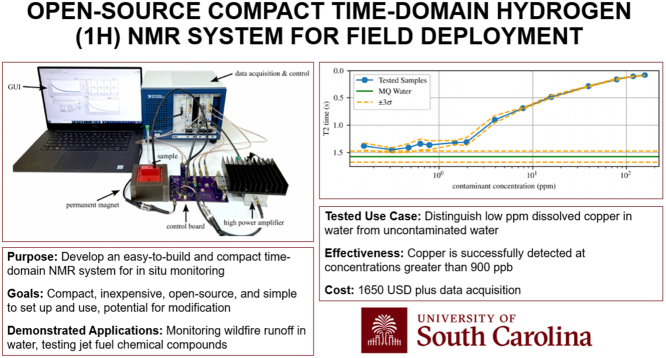

**Table:** 

**Specifications table**	

Hardware name	Bench Top Nuclear Magnetic Resonance Open-Source Compact Time-Domain Hydrogen (1H) NMR System for Field Deployment

Subject area	• *General* • *Environmental, planetary and agricultural sciences*

Hardware type	• Field measurements and sensors

Closest commercial analog	Niumag Time domain NMR Instrument

Open source license	CC BY-SA 4.0

Cost of hardware	1650 USD plus data acquisition system

Source file repository	https://doi.org/10.17605/OSF.IO/ND27H

	

## 1 Hardware in context

**Fig. 1:**
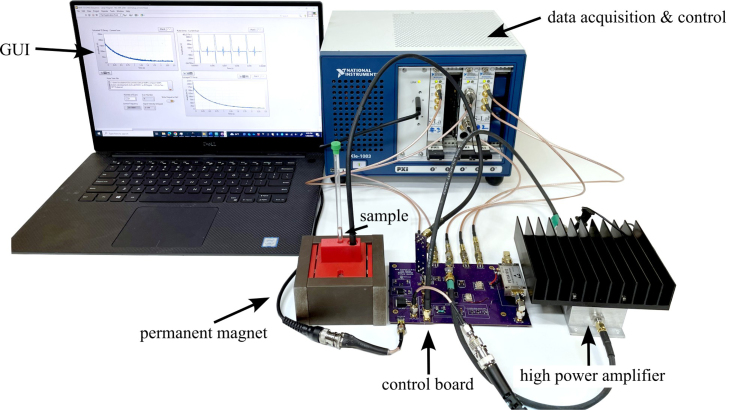
Full setup for the compact TD-NMR system, with key components and subsystems annotated.

Our compact open-source time-domain NMR (TD-NMR) system [1] (shown in Fig. 1) addresses the limitations of traditional high-field Nuclear Magnetic Resonance (NMR) by offering a versatile, low-cost, and portable solution specifically for hydrogen (^1^H) NMR applications. Unlike traditional NMR systems, which often require liquid helium-cooled superconducting magnets and extensive infrastructure, our TD-NMR utilizes permanent magnets and simplified electronics, enabling easy operation in field environments. This portability and affordability make it well-suited for applications such as fuel quality analysis [2], [3], environmental monitoring [4], [5], and other in-situ assessments such as online process control or material characterization. By focusing on hydrogen NMR, our system maximizes sensitivity and accessibility, providing a practical alternative to high-field systems for diverse scientific and applied research needs outside of conventional lab settings.

NMR spectroscopy is widely used for its ability to non-invasively reveal molecular structures and compositions, often through the study of hydrogen atoms [6]. By applying radiofrequency (RF) pulses in a magnetic field, NMR spectroscopy provides insights into chemical environments and molecular interactions, making it a valuable tool across chemistry, biology, and material science. While traditional high-field NMR systems offer exceptional resolution, they are costly, require specialized infrastructure, and are generally confined to laboratory settings.

Beyond spectroscopy, NMR relaxometry is another important application that measures the relaxation times ($T_{1}$ and $T_{2}$) of nuclei returning to equilibrium after excitation [7]. Each relaxation time is an exponential decay constant that, when measured, can be used to extract properties of the sample such as molecular motion [8] and material composition [9]. For in-depth information about the physics involved with NMR relaxometry, please consult the text by Macomber [10]. $T_{2}$ relaxation involves an important parameter called the Larmor frequency, which is a side-effect of quantum mechanics and angular momentum, described by:

(1) $$\omega_{0}=B_{0}\gamma ,$$

where $\omega_{0}$ (MHz) is the Larmor frequency, $B_{0}$ (T) is the external magnetic field strength, and γ (MHz/T) is the gyromagnetic ratio. The $T_{2}$ relaxation time can be measured by applying a magnetic field oscillating at the Larmor frequency [11].

Relaxometry primarily focuses on hydrogen (^1^H) nuclei due to their abundance in organic and biological materials, high natural abundance, and strong signal response, making them especially suited for studying water content, viscosity, and tissue characteristics. However, NMR relaxometry is also applicable to other nuclei, such as carbon (^13^C), phosphorus (^31^P), and fluorine (^19^F) [12], each offering unique insights based on their nuclear properties and the specific environments in which they are found.

Magnetic Resonance Imaging (MRI), which is based on the principles of NMR, uses the relaxation properties of nuclei to generate detailed images of soft tissue. Traditionally, MRI systems require large, high-field magnets and extensive infrastructure, limiting their use to clinical environments. However, recent advancements in portable, low-field MRI, such as point-of-care systems utilizing ultralow-field permanent magnets [13], have dramatically reduced these requirements. These developments increase accessibility in medical imaging while increased component availability also enables the creation of compact, low-cost NMR systems for field applications like environmental monitoring and fuel analysis.

Compact NMR has become an established field [7], driven by advances in magnet design [14] that allow permanent magnets to achieve the homogeneity needed for applications previously restricted to high-field systems [15]. While high-field NMR remains indispensable for tasks requiring extremely high resolution, compact low-field NMR systems now offer a practical, cost-effective alternative for routine analysis and field applications. These systems, often referred to as desktop or mobile NMR [16], enable multi-nuclear and multi-dimensional NMR relaxometry or spectroscopy on the benchtop, making them accessible for chemical analysis, reaction monitoring, and nondestructive evaluation. With applications ranging from food industry quality control [17] to the monitoring of oil wells [18], compact NMR [19] instruments have proven valuable in diverse settings. Their portability and ease of use have opened up new possibilities for real-time, on-site analysis in fields like environmental monitoring [4] and process control [20], without the need for the expensive infrastructure or maintenance associated with high-field NMR.

Various open-source NMR systems have emerged, each tailored to specific applications. For example, the NMRduino platform by Tayler and Bodenstedt emphasizes accessibility for educational purposes and simplicity in hardware setup, making it ideal for users seeking to understand basic NMR principles at low cost [21]. However, its limited frequency range restricts its ability to resolve fine chemical shifts, making it less suited for advanced applications requiring higher sensitivity. Similarly, Louis-Joseph and Lesot’s FT-NMR system is a low-cost benchtop spectrometer optimized for teaching and basic molecular analysis [22]. While cost-effective, this system’s benchtop focus limits its applicability to field studies. In contrast, Bryden et al. developed an ultra-low-field (ULF) NMR system featuring RF mixing and pre-polarization, enhancing signal strength and resolution, making it well-suited for hyperpolarized gas detection [23]. However, its low field strength may limit sensitivity for applications requiring high spectral resolution. Our compact time-domain NMR system builds on these principles but extends functionality with a focus on versatility and field deployment. Designed for robustness in demanding environments, it supports higher operational frequencies and automatic tuning, enabling use by non-expert users for in-situ applications like environmental monitoring and fuel analysis. While previous systems cater to specific educational or niche scientific needs, our system’s modular open-source design allows for extensive customization, making it suitable for a wide range of research applications requiring reliable NMR measurements beyond the lab.

In previous studies, the TD-NMR system developed in this work Fig. 1 has proven its adaptability and utility across multiple contexts. Initially, it was employed to measure hydrogen content in gas turbine fuels, where it provided rapid and accurate determinations of hydrogen mass percentages, crucial for evaluating combustion properties [2]. The system has also been utilized to assess cetane numbers in jet fuels. By analyzing $T_{2}$ relaxation curves, the system predicts the derived cetane number with high accuracy, enabling real-time fuel quality assessments that traditional ASTM standards would typically handle in a more time-consuming manner [3] Subsequently, the system was adapted to track magnetic particle concentrations in wildfire ashes and runoff [4] and quantifying algae uptake of heavy metals [5] highlighting its effectiveness in environmental monitoring scenarios.

The adaptability of this compact TD-NMR system across diverse applications underscores its potential as a valuable tool for both research and practical deployment. This system has already been adapted for use in in-situ remote deployment [24]. Its open-source nature allows users to tailor the system to specific needs, making it a cost-effective and scalable solution across various scientific disciplines. The contributions of this hardware are:

1. The first open-source, low-cost NMR system that combines portability, permanent magnets, and customizable electronics, specifically designed for field-deployable, in-situ measurements. Unlike previous designs, this system uniquely enables real-time monitoring outside of traditional laboratory environments.
2. A well-developed and extensively validated system, having demonstrated its efficacy in multiple peer-reviewed scientific publications across a range of applications. This includes studies on fuel characterization, magnetic particle quantification in environmental samples, and algae analysis.

## 2 Hardware description

This section describes the hardware, software, and sufficient theory required for the reader to understand the developed open-source compact TD-NMR system.

### 2.1 Overview

The NMR system was developed with a foundation based on a custom printed circuit board (PCB) for all the analog signal processing and an off-the-shelf data acquisition and control system built around PXI cards from National Instruments and controlled through a custom LabVIEW code, as shown in Fig. 1. The use of an off-the-shelf data acquisition was done to minimize the challenges and intricacies involved in developing a precise signal generation and data acquisition system. If properly designed, a custom data acquisition and control system could replace the PXI-based system, greatly reducing overall cost. The compact-NMR uses the NI PXIe-1083 chassis with three modules. These include two NI PXI-5421 cards for signal generation and an NI PXI-5124 card for triggering and collecting the signals. Data is transferred back to the host computer using a Thunderbolt connection. While contemporary off-the-shelf NMR solutions offer exceptional precision in measurements and are meticulously calibrated to enable high-resolution assessments, they are typically configured to operate at fixed frequencies without the flexibility of adjustment. The system presented in this work allows for precise data to be collected and with simple adjustments in the LabVIEW code, it is possible to change frequency, timing, and manipulation of the signal generation. This enables users to modify the system, according to their own specific needs. Overall, the system was designed with the following goals in mind:

• Generate clear time domain $T_{2}$ NMR results in under 60 s
• Function as a compact desktop NMR system.
• Be easily replicated through the absence of shim coils.
• Easily be adapted into a field deployable NMR system.

**Fig. 2:**
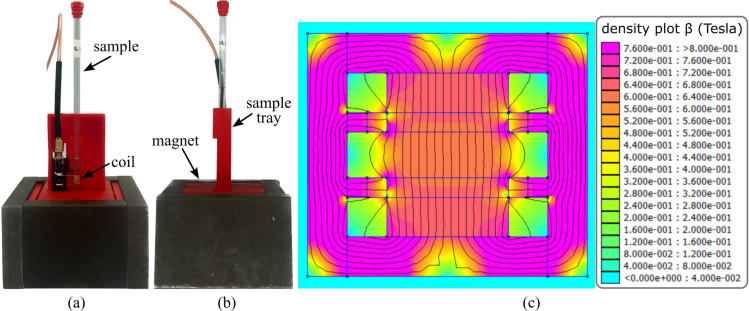
Magnet overview, showing: (a) Front view of assembled magnet configuration with tray and coil; (b) side view of assembled magnet configuration and (c) two-dimensional finite element simulation [25].

### 2.2 Magnet design

Magnet design for compact NMR is a constantly progressing field that has developed a large number of magnets suitable for use in compact NMR systems. For example, Moresi and Magin developed a miniature permanent magnet for NMR applications capable of achieving a high homogeneity (10 ppm over a $3\times 3\times 5\;{\mathrm{mm}}^{3}$ volume) at 0.6 T, scalable up to 3 T through additional magnetic layers, suitable for portable field-deployable NMR systems [26]. Polishchuk and Gardeniers introduced a compact permanent magnet for NMR relaxometry with a novel integration of soft-magnetic stainless-steel plates in the airgap, significantly enhancing field homogeneity for microfluidic flow measurements [27]. Raich and Blümler developed a dipolar Halbach array utilizing identical bar magnets to create a homogeneous magnetic field, optimizing the array for mobile NMR devices by balancing field strength, homogeneity, and weight [28]. Alnajjar et al. utilized 3D printing with a stainless steel-PLA composite, to develop lightweight, high-performance NMR magnets for portable applications [15].

Homogeneity of the magnetic field is crucial in the design of compact NMR magnets. The more uniform the magnetic field, the higher the quality of the NMR signal, enhancing signal clarity and accuracy [14]. However, this uniformity narrows the bandwidth of frequencies that effectively excite the sample, necessitating a more precisely calibrated excitation signal. Additionally, environmental temperature changes can impact the magnet’s strength, a significant consideration for compact NMR systems used in field applications. These variations can make tracking the excitation bandwidth challenging in magnets with highly homogeneous fields. In brief, high homogeneity results in better signal strength but comes at the cost of overall signal robustness, and this trade-off in magnet design is a variable that should be considered by system developers.

The design presented in this work is a center-field magnet with a steel housing and steel pole shoes [29]. This design was chosen as it is relatively low-cost, easy to reproduce, the steel housing constrains the magnetic field making the magnet assembly easier to handle once assembled. The magnet assembly features two main N42 neodymium-iron-boron (NdFeB) magnets with dimensions of 3.81×3.81×1.27 cm as well as eight aligning magnets made of the same material with dimensions of 3.81×0.635×0.635 cm. These aligning magnets make the magnet stronger and greatly increase homogeneity. 1018 steel is used to provide a return path for the magnetic flux, making the magnet stronger and significantly safer to handle [4]. The developed permanent magnet is shown in Fig. 2 and has a Larmor frequency of 24 MHz ±2% while maintaining a small footprint of 90×80×50mm with a protective metal casing. When assembled, but without the tray or coil, the magnet weighs 1.99 kg. Fig. 2(a) and (b) depict the fully assembled magnet. Fig. 2(c) shows a 2D Finite Element Method Magnetics (FEMM) [25] simulation of the magnet design. It shows how the 1018 steel bars provide a pathway for magnetic flux as well as depicting the field strength of the magnet. The field strength in the center of the magnet can differ by approximately ±0.2 T according to the simulation, providing an estimation of field inhomogeneity.

### 2.3 Pulse sequence

**Fig. 3:**
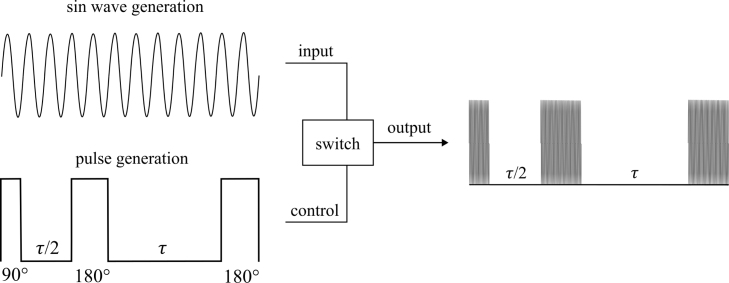
The CPMG technique where a square pulse wave acts as the control for the switch, while the sin wave acts as the input. When the square wave is high it activates the switch letting the sin wave travel through creating a pulsed sine wave.

**Fig. 4:**
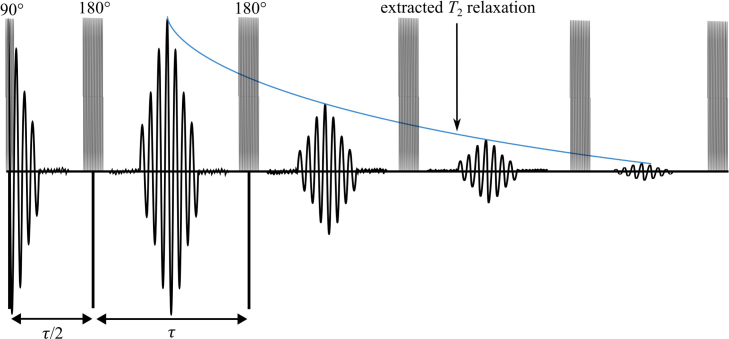
Expected response of the spin Echoes. The CPMG sequence includes a 90-degree pulse followed by a downtime τ/2 then continuous 180-degree pulses followed by downtimes of τ.

Due to the relatively low strength and high inhomogeneities of the magnet used in this work, both single pulse and chemical shift NMR are not possible. To remedy this, this system uses the Carr-Purcell-Meiboom-Gill (CPMG) technique [30]. The CPMG technique involves a precise pulse sequence that is visualized in Fig. 3. In the implementation process, the system applies a magnetic pulse to the sample that rotates the nuclear spins by 90 degrees around an axis perpendicular to the field of the permanent magnet. This pulse is referred to as the 90 degree pulse. Subsequently, a continuous sequence of magnetic pulses is applied to the sample that rotate the nuclear spins by 180 degrees around an axis perpendicular to the field of the permanent magnet. These will be referred to as 180 degree pulses. Immediately after the 90 degree pulse, the nuclei will be fully aligned perpendicular to the applied field from the magnet. Shortly after, this alignment begins to dephase. While the alignment is dephasing, a 180 degree pulse is applied. Since the rotation of the 180 degree pulse is around an axis perpendicular to the field of the permanent magnet, this causes the spins to flip direction. If they were up, they are now down and if they were down they are now up. Because of this and conservation of angular momentum, rather than continuing to dephase, the nuclear spins rephase. Furthermore, the magnitude of the spins remain the same so the time it takes the spins to rephase will be the same amount of time that it took them to dephase. This resonance effect is called a spin echo and can be measured. An example of spin echoes can be seen in Fig. 4. After being aligned, the nuclear spins become unaligned again in the exact same way they did after the initial 90 degree pulse. By continuing the 180 degree pulses with the same time interval between them, the spin echoes can be measured throughout the relaxation process, until eventually all of the nuclear spins are back to where they were before the 90 degree pulse. Taking the highest point in each spin echo allows for a $T_{2}$ relaxation curve to be constructed. In this method, the NMR system can produce clear resolution time domain results while maintaining a small compact footprint that, unlike other NMR systems, does not require any shim coils [31] or complicated calibration to achieve.

**Fig. 5:**
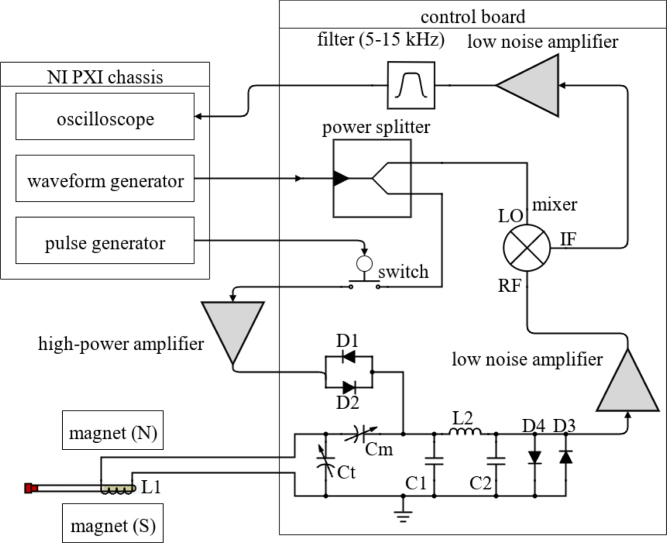
Schematic of the custom electronics used for the compact NMR system.

### 2.4 Custom electronics

Fig. 5 shows a schematic of the developed electronics consisting of two main parts, the custom-developed control bard and the off-the-shelf PXI-based data acquisition system. On the control board, the switch is used to control the pulses that excite the sample. When the pulse generator sends a signal into the switch, the sine wave is able to travel through the switch creating a pulsed signal. The creation of the pulsed sine wave is visualized in Fig. 3 The signal then travels through an amplifier and into the coil. Subsequently, the resonance from the sample is directed through an additional amplifier to enhance the signal under collection. It should be noted that amplifiers with lower gain have been tested, providing mixed results. From this point, the signal is routed to a mixer to fine-tune the frequency to approximately 15 kHz, facilitating its passage through a filter and a low-noise amplifier. This sequence serves to diminish extraneous noise and unveil the distinct resonance signal. The duplexer is used to make sure only the nuclei resonance travels through the amplifier and to block any of the signals from traveling back through the system. The voltage regulators that are used include a 12, 5, and 1.8 V regulator. These components are integrated to streamline the system into a single 24 V power supply requirement, aimed at delivering approximately 1 A of current. Finally, a separate impedance matching board is used for calibrating and tuning the system and is described in-depth in Section 5.2. Information about the data acquisition system can be found on the specifications sheet for the PXI-5124 digitizer. At a 50 Ω impedance, there are two filters that can be applied exclusively, a 20 MHz 2-pole Bessel filter for noise filtration and a 60 MHz 4-pole elliptical filter for anti-aliasing. As a default, only the anti-aliasing filter is on. The dynamic range at a 50 Ω impedance varies, decreasing from 75 DBc at 0.2 V to 65 DBc at 10 V [32].

### 2.5 Software

The software of the compact-NMR system is written in LabVIEW. The code controls the wave generation and data collection. The pulse generator functions by making an arbitrary waveform controlled by a pulse train file, turning it on and off. The sine wave generates a consistent frequency which can be set to any frequency in between 0–43 MHz. The code sets the timing and triggers to assure consistent phase of the excitation pulse. The code finally collects the data and plots the peaks of the spin echoes to produce a $T_{2}$ decay curve. A number of scans can also be set to get a higher resolution result by averaging out the results from each scan; as is typically done with this system.

### 2.6 Safety and regulatory information

As this is a magnetic resonance system, it is subject to certain US federal regulations. The frequency of this system is permitted by these regulations and the power of the system is in the range of −20 to −30 dBm depending on the specific calibration, which is well below 1 mW. Due to this, the field strength is also well within regulatory requirements. As the system will need to be troubleshooted during operation, always ensure that the power supply is turned off when not in use. While the system operates at a maximum of 24 V, shocks are unlikely, but possible when not operating safely. According to OSHA regulations, while low-voltage systems, (typically below 50 V) present a reduced risk of electric shock, they can still pose hazards such as short circuits, burns, or risks in wet environments [33].

## 3 Design files summary

Table 1 reports a summary of the major design files with a description provided below.

• Control Board PCB: This circuit board is the main board of the system and was developed in KiCAD.
• Impedance Matching PCB: This circuit board is used to ensure that the electronics operate at the Larmor frequency and was developed in KiCAD.
• Coil PCB: This PCB holds the coil that creates a small magnetic field to cause an NMR in the sample.
• Magnet Tray: This STEP file is for the tray that holds the coil PCB in the magnet.
• Magnet Casing: This STEP file is for holding the magnets in place during and after the construction of the magnet.
• NMR T2 CPMG Sequence.vi: This is a LabVIEW VI file that takes data and calculates $T_{2}$ curves.
• NMR Single Shot Pulse Sequence.vi: This is a LabVIEW VI file that is used for tuning the Larmor frequency.
• 7μs 90 Deg-1.25 ms Tau-4 Pulses.txt: This is a file that contains a short pulse sequence to use with <NMR Single Shot Pulse Sequence.vi> for Larmor frequency tuning.
• 7μs 90 Degree - 0.7 ms Tau - 5000 Pulses.txt: This is a file that contains a long pulse sequence to use with the CPMG Sequence VI for taking data and generating $T_{2}$ curves.
• pulse_sequence_creator_PXI5421.ipynb: This is a MATLAB file that can be used to generate new pulse sequences.

**Table 1:** Table of design files.

Design filename	File type	Open source license	Location of the file
Control Board PCB	EDA file	CC BY-SA 4.0	https://doi.org/10.17605/-OSF.IO/ND27H/
Impedance Matching PCB	EDA file	CC BY-SA 4.0	https://doi.org/10.17605/-OSF.IO/ND27H/
Coil PCB	EDA file	CC BY-SA 4.0	https://doi.org/10.17605/-OSF.IO/ND27H/
Magnet Tray	STEP file	CC BY-SA 4.0	https://doi.org/10.17605/-OSF.IO/ND27H/
Magnet casing	STEP file	CC BY-SA 4.0	https://doi.org/10.17605/-OSF.IO/ND27H/
NMR T2 CPMG Sequence	VI file	CC BY-SA 4.0	https://doi.org/10.17605/-OSF.IO/ND27H/
NMR Single Shot Pulse Sequence	VI file	CC BY-SA 4.0	https://doi.org/10.17605/-OSF.IO/ND27H/
7 μs 90 Deg-1.25 ms Tau-4 Pulses	TXT file	CC BY-SA 4.0	https://doi.org/10.17605/-OSF.IO/ND27H/
7 μs 90 Degree - 0.7 ms Tau - 5000 Pulses	TXT file	CC BY-SA 4.0	https://doi.org/10.17605/-OSF.IO/ND27H/
pulse_sequence_creator_PXI5421	Jupyter Notebook	CC BY-SA 4.0	https://doi.org/10.17605/-OSF.IO/ND27H/

## 4 Bill of materials summary

A complete bill of materials is located at https://doi.org/10.17605/OSF.IO/ND27H/.

## 5 Build instructions

This section outlines the build instructions for the NMR system.

### 5.1 NMR assembly

The base NMR system is built in three stages. First, the magnet housing must be assembled which includes 3D printed parts and (1018 steel bars). Second, the PCBs must be assembled with the proper electronic components. Third, the system needs to be calibrated to the correct frequency.

Tools used for the construction of the package include the following:

• Soldering iron
• Sn60/Pb40 22 gauge solder
• Sn63/Pb37 solder paste
• Flathead screwdriver
• Pliers
• Razor blade
• Instant glue (i.e. Cyanoacrylate Adhesive or Super Glue®)
• Clamps
• Wood slats

Other optional tools

• Re-flow soldering bed

#### 5.1.1 Building the magnet

**Handle magnets with extreme caution! Keep away from metal objects and other magnets. Gloves highly encouraged**

**Fig. 6:**
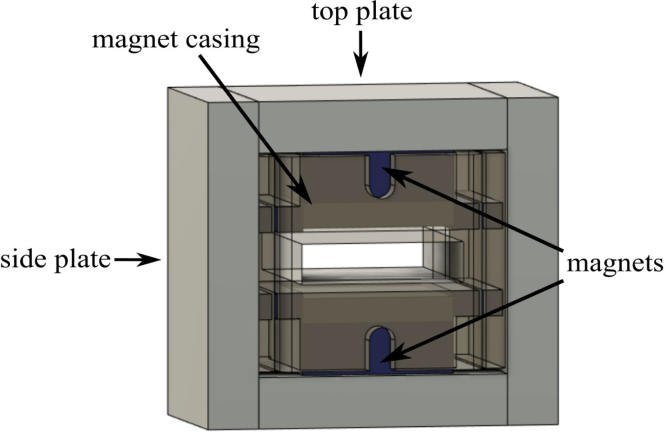
Fully assembled magnet.

The process for building and assembling the main magnet shown in Fig. 6 is described in the enumerated steps below.

**Fig. 7:**
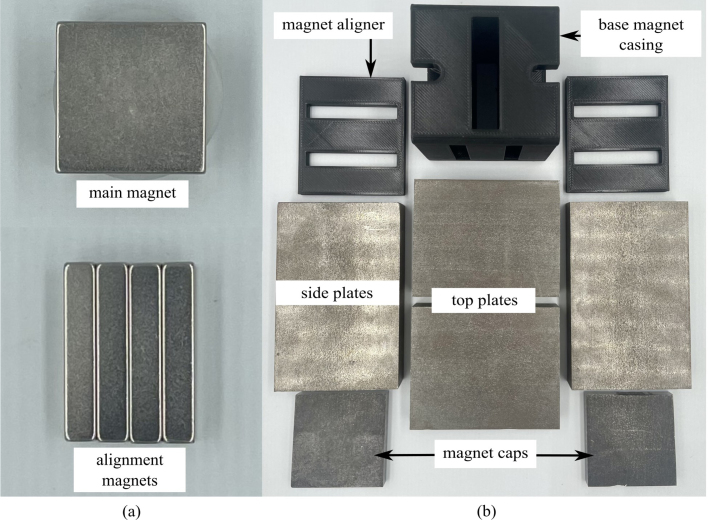
Constituent parts for the magnet assembly, showing: (a) magnets, and (b) steel field guides and plastic component holders.

**Fig. 8:**
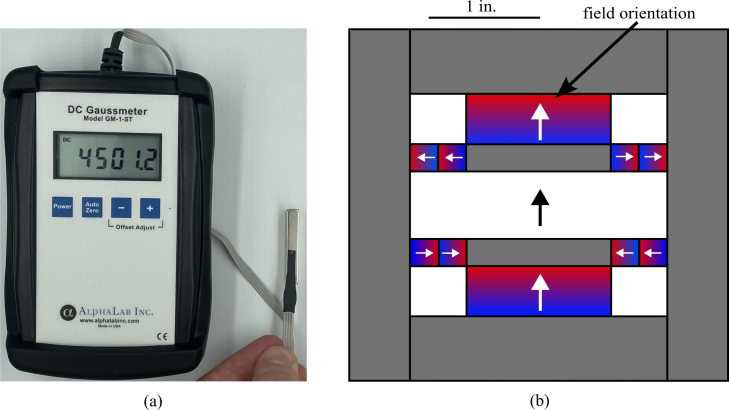
Measuring magnet polarity, showing: (a) Gaussmeter measuring an alignment magnet in the positive magnetic field orientation, and (b) a diagram showing polarities of the magnetic assembly as well as a size reference.

**Fig. 9:**
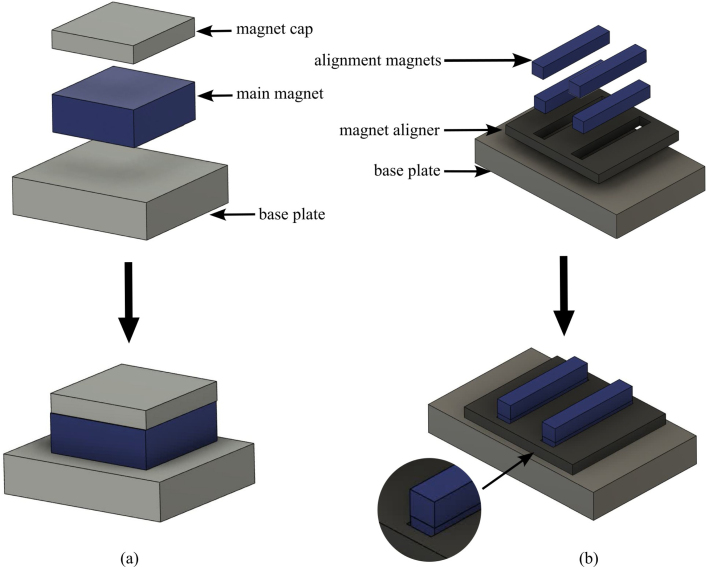
Magnetic component assembly, showing: (a) construction of sub-assembly a the top plate to the main magnet and steel magnet cap, and (b) sub-assembly b gluing the magnet aligners to the side plate and inserting the alignment magnets.

**Fig. 10:**
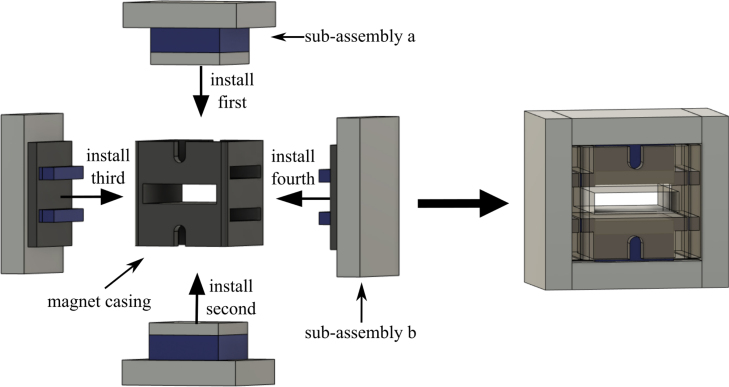
Magnetic assembly, showing the order of operations for adding the side assemblies to the plastic frame.

1. Obtain materials in Table 2. Fig. 7 presents an image of these components.
2. Once materials for the magnet have been obtained, it is time for assembly. Fig. 8 provides an overview of the correct polarity orientation of the main and aliment magnets. Refer to Fig. 9, Fig. 10 for visualizations. **Reminder magnets are extremely dangerous and need to be handled with planning and caution.**
3. For sub-assembly a (Fig. 9(a)), begin by attaching the main magnets to steel magnetic caps (make sure each sub-assembly a has opposite polarities using a Guassmeter). Next, attach the main magnets with steel to the center of each top plate. It is best to use layers of non-magnetic slats (such as wood or plastic) in between the magnets and steel to avoid sudden attraction that could lead to damage to the magnet or injury to yourself. Slowly remove layers of non-magnetic slats until the magnet is flush with the steel.
4. To begin assembling sub-assembly b (Fig. 9(b)), instant glue the magnet aligners to the center of each side plate with the magnet slots parallel to the short edge of the side plate and put them aside for later.
5. Next for sub-assembly b, take two of the alignment magnets and insert them into each of the magnet holders. Make sure to pay attention to the polarity of the magnets using a Gaussmeter and follow the polarities shown in Fig. 8. Note that the Guassmeter’s probe needs to stay in the same orientation while measuring polarity as it will cause the reading to change signs if the opposite side of the probe is used.
6. Next insert sub-assembly a into one side of the magnet casing. Make sure the steel bar is flush with the top of the magnet casing. Refer to Fig. 10.
7. Carefully insert the second sub-assembly a into the other side of the magnet casing.
8. Once the magnets are correctly positioned and the polarities are in the correct orientation, carefully bring the first sub-assembly b edge in contact with the ends of the top plates and slowly let it become flush with them. Note that this side should have a very strong attraction to the center of the magnet and should go on easily with the magnets in the correct polarity.
9. Take the second sub-assembly (b) and bring it into contact with the other ends of the top plates. This time, applying force will be necessary to ensure it is flush and securely attached to the rest of the magnet, provided the polarities are correctly aligned.
10. Measure the inductance of the assembled magnet. This value should be close to 0.56 T. Refer to Section 5.2. If the value is measurably less than 0.56 T, then the polarity orientation of the magnets may be incorrect.

**Table 2:** List of parts required for magnet assembly.

Amount	Description	Part name	Location
1X	3D printed magnet casing	Magnet casing	https://doi.org/10.17605/-OSF.IO/ND27H/
2X	3D printed magnet aligner	Magnet aligner	https://doi.org/10.17605/-OSF.IO/ND27H/
2X	1018 carbon steel 12.7×50.8×64 mm	Top plate	Bill of materials
2X	1018 carbon steel 12.7×50.8×80 mm	Side plate	Bill of materials
2X	1018 carbon steel 38.1×38.1×6 mm	Magnet cap	Bill of materials
2X	BX8X88 magnet	Main magnet	Bill of materials
8X	BX844 magnet	Alignment magnet	Bill of materials

#### 5.1.2 Assembling the PCB

**Fig. 11:**
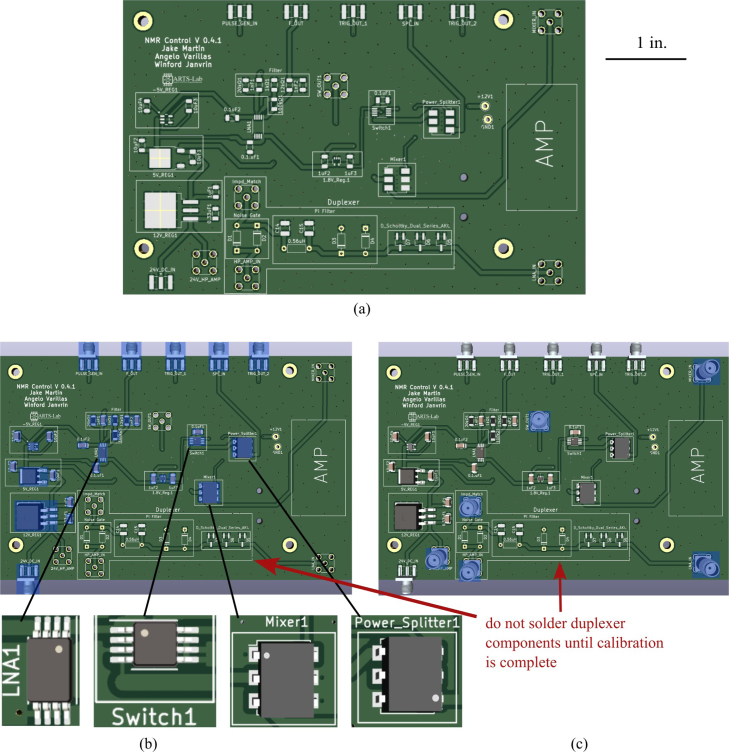
Control board PCB assembly, showing: (a) control board and size reference, (b) soldered SMA components including orientation of components, and (c) soldered through hole components.

**Fig. 12:**
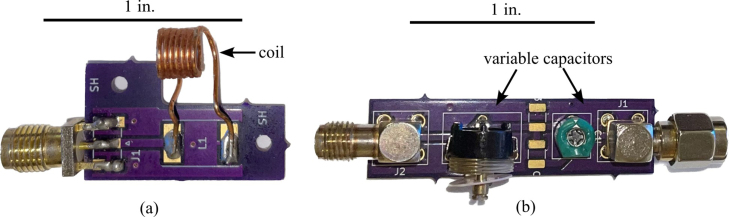
Component sub-boards, showing: (a) coil PCB. (b) populated impedance matching PCB as well as size references for each component.

This section outlines the detailed procedure for assembling the printed circuit boards (PCBs) critical for the NMR system’s operation, ensuring the correct order of component placement/soldering is essential as the duplexer should not be installed till calibration is complete. The assembly steps are:

1. Begin by obtaining the NMR Control Board, Impedance Matching, and Coil PCBs provided from the Electronic Design Automation (EDA) files in the design files summary.
2. Obtain the SMD components including the Mixer, Switch, Power Splitter, Low Noise Amplifier, 1.8 V regulator, 5 V regulator, 12 V regulator, Charge Pump Converter, Resistors, and Capacitors. Values are listed on the PCB. Specific parts can be found in the bill of materials Section 4.
3. Start by soldering the SMD components on the Control board, excluding those in the duplexer section (the diodes, inductor, and two capacitors). This does include the surface mount SMA connectors. Apply solder paste and place the components on their respective pads. Use a reflow soldering oven set to 400–425 °C or an equivalent method. Avoid using a heat gun as it may melt and damage the components. Ensure that you follow the resistor and capacitor values marked on the board. Refer to Fig. 11 for the correct orientation of components, where dots on each component indicate pin 1.
4. Next, solder the through-hole SMA connectors to the board.
5. Once soldering is complete check for continuity and shorts between the pins of each component. To check for continuity, follow the traces on the PCB denoting wires from component to component. Grounded vias are present throughout the board to aid in checking for shorts.
6. The next step is assembling the coil PCB. To accomplish this, utilize a cylinder (such as a screwdriver) with a 5 mm diameter, matching that of the glass tube, take 140 mm of copper wire, and precisely wind the wire around seven times. It is essential to maintain a tight wire, minimizing any gaps between each coil. Tweezers can be employed to achieve this desired outcome, followed by the utilization of instant glue to secure the coil in place. Refer to Fig. 12(a) for the final outcome.
7. Moving on to the impedance matching board, solder the variable capacitors and SMA connectors to their respective holes. Fig. 12(b) shows completed PCB.
8. Once the instant glue has set and the coil is finished solder the two ends onto the PCB.
9. Lastly is the duplexer section of the Control PCB, this board requires calibration so follow the steps in order. First solder the SMA connectors and the inductor (0.56 μH) to their respective spots. Once done Move to Section 5.2 to finish calibrating and soldering of the PCB.

**Fig. 13:**
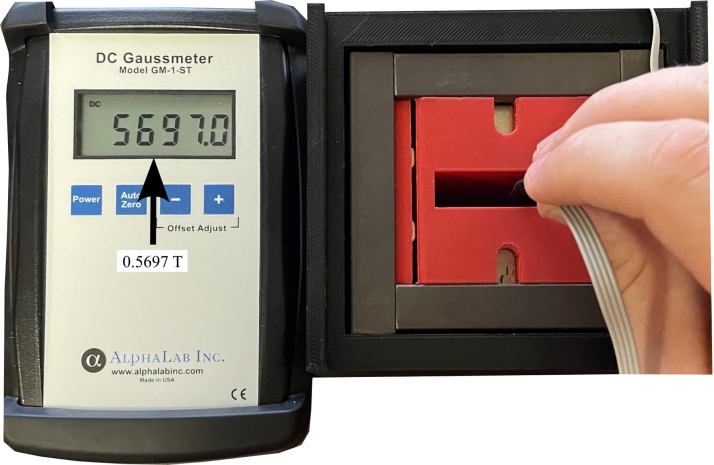
Recording magnet inductance using a Gaussmeter.

### 5.2 Probe calibration

**Fig. 14:**
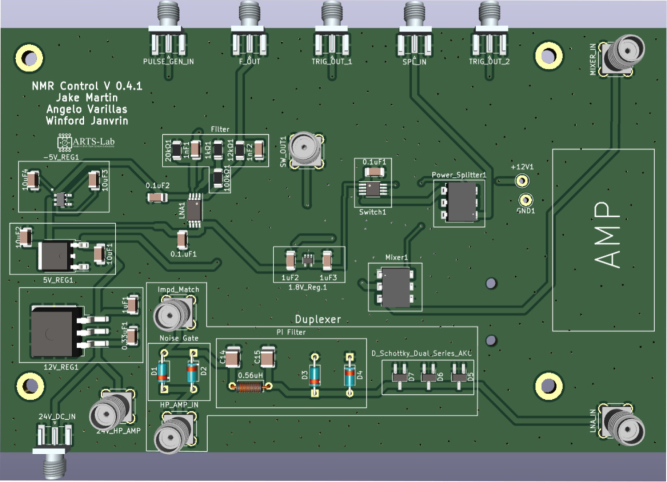
Populated control board.

**Fig. 15:**
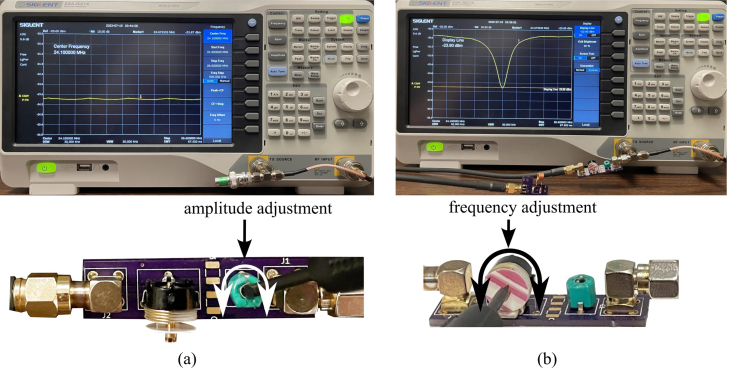
Calibration of the probe, showing: (a) setting the display line at 50 Ω feed-through terminator signal and (b) properly calibrated impedance matching board and coil.

**Fig. 16:**
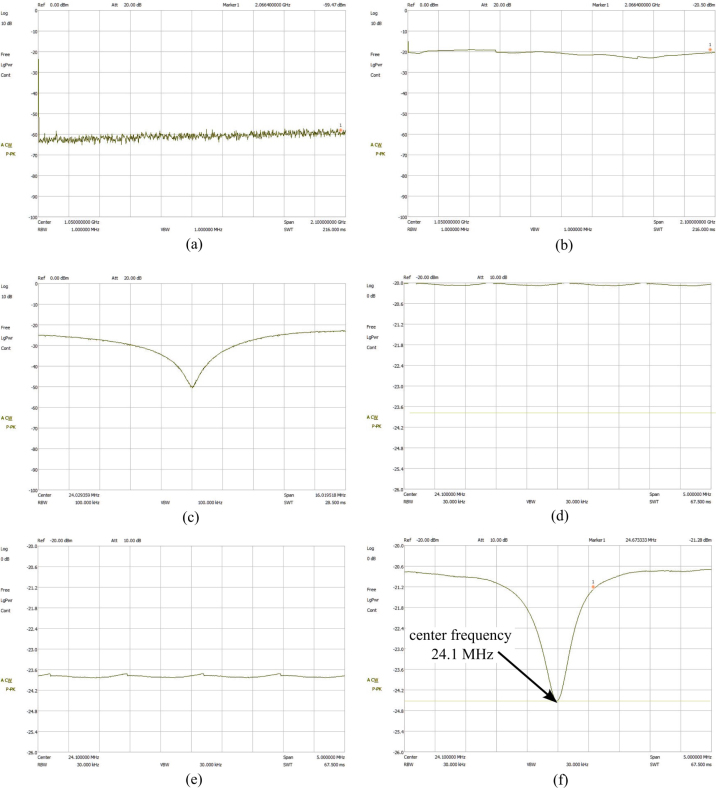
Spectrum analyzer setup and calibration of impedance matching board and duplexer showing: (a) spectrum analyzer default settings, (b) tracking generator setting turned on and level set to −20 dBm, (c) frequency adjusted and calibrated duplexer board connected, (d) frequency, span, and amplitude settings adjusted to calibrate impedance matching board, (e) 50 Ω feedback terminator connected to create reference line, and (f) properly calibrated impedance matching board connected to spectrum analyzer and coil PCB.

This section is for calibrating the coil/probe and pi filter using the impedance matching and Duplexer boards. Note this only needs to be done once during assembly but can also be used for troubleshooting the system. The steps to calibrate the probe are:

1. Begin by acquiring a Gaussmeter. Once acquired, measure the inductance of your magnet by inserting it at the very center (refer to Fig. 13). Record the highest value in Tesla.
2. To calculate the Larmor frequency $\left(\omega_{0}\right)$ of your magnet use the following equation
  (2) $$\omega_{0}=B_{0}\cdot 42.58,$$
  where $\omega_{0}$ (MHz) is the Larmor frequency, $B_{0}$ (T) is the magnet inductance and 42.58 (MHz/T) is the gyromagnetic constant for hydrogen. The result should be in the low to mid 20 MHz. (Our Larmor frequency was 24.2 MHz)
3. With this Larmor frequency we are now able to calculate an approximate capacitor value for the duplexer. Since this is an LC circuit, the angular frequency of the circuit can be found using:
  (3) $$\omega_{0}=1/\sqrt{LC}.$$
  Solving for C(F) results in:
  (4) $$C=\frac{1}{{\left(f_{0}\cdot 2\pi \right)}^{2}\cdot L},$$
  where C (F) is the capacitance value, $f_{0}$ (Hz) is the Larmor frequency, and L (H). Here, the extra factor of $1/{\left(2\pi \right)}^{2}$ comes from the Larmor frequency being used as the linear frequency of a sine wave in the LC circuit. The calculated capacitance value should be between 65 pF and 85 pF. Since every magnet will not have the exact same frequency, this is reported as a range. Additionally, the measured calibration can differ from this ideal calculation. In testing, C15 controlled the center frequency of calibration and C14 helped to regulate ripple. For this system, C15 was 60 pF and C14 was 68 pF.
4. Solder calculated capacitors to the duplexer board.
5. Obtain a spectrum analyzer and a BNC 50 Ω feed-through terminator to use for the calibration.
6. Connect the tracking generator source to the RF input.
7. Turn on the spectrum analyzer and go to the tracking generator menu. In the menu set the level to −20 dBm.
8. Open the frequency menu and set the center frequency to the calculated Larmor frequency.
9. Open the span menu and adjust the span to 30 MHz.
10. Connect the Imped Match SMA on the NMR Control Board to the spectrum analyzer. A dip should appear near the Larmor frequency of the magnet. If the dip is not within ±1 MHz of the frequency then a different capacitor value will need to be chosen and soldered on the board. This will need to be repeated until this criteria is met.
11. Once the duplexer is properly calibrated, solder on the diodes. This will complete the control board represented in Fig. 14.
12. Next, the impedance matching board and coil will be calibrated. Refer to Fig. 15, Fig. 16 for visuals.
13. Continue by opening the amplitude menu and setting the reference to −20 dBm and the scale to 0.6 dB.
14. Open the span menu and set the span to 5 MHz.
15. Plug in the 50 Ω feed-through terminator.
16. Create a display line over the signal that will be used as a reference later.
17. Connect the impedance-matching PCB to the coil PCB using a male BNC cord. Note that changing this chord later can affect the calibration. It should be noted that the specific variable capacitors are intended to be used with cords between 6 and 30 in. Cord lengths outside this range will require different variable capacitors. It is also important to use a high-quality cord for this. The bending of the cord during calibration and in the fully assembled system will be different, causing calibration issues with low-quality cords.
18. Now remove the 50 Ω feed-through terminator and attach the impedance-matching PCB with the coil PCB. A dip should be visible on the screen.
19. Use a small flathead screwdriver to adjust the variable capacitors until the very center of the dip is at the Larmor frequency and touching the reference display line.

### 5.3 Final assembly

This subsection provides a comprehensive guide for the final assembly process of the NMR system, detailing the integration and connection of all components to ensure operational readiness for field deployment.

**Fig. 17:**
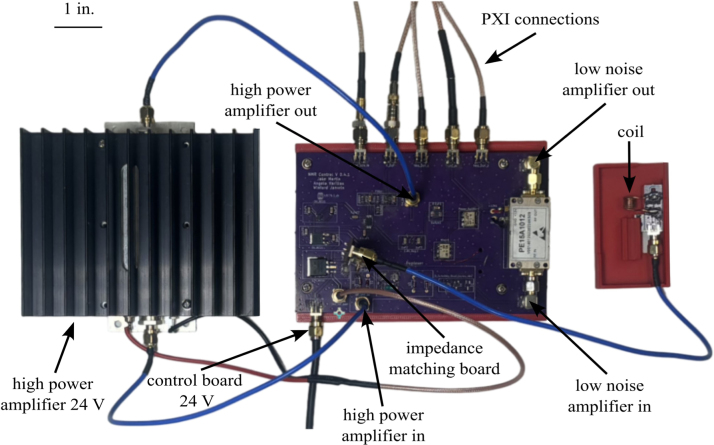
Control board with amplifieres, wiring, and size reference.

**Fig. 18:**
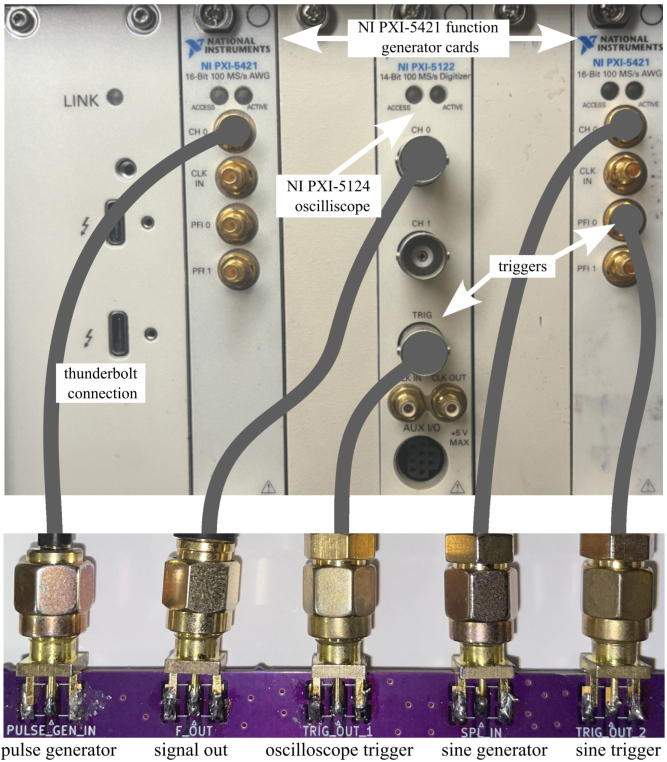
Wiring diagram from the PXI cards to the control board.

**Fig. 19:**
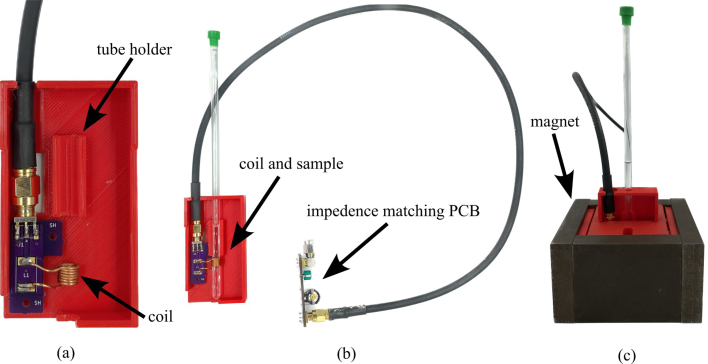
Details of the sample tube and coil tray, showing: (a) insert the coil PCB into the magnet tray; (b) complete assembly of coil and tray with sample, and; (c) tray with sample inserted in magnet.

1. To begin the final assembly first acquire an NI PXIe chassis or equivalent system for waveform generation and data collection. There are a number of PXI cards available form National Instruments or other vendors that could be used for this purpose. In general, this hardware requires a digitizer and two independent channels of arbitrary waveform generators (AWG).
2. Using hardware available to the authors, a PXIe-1083 chassis and three PXI cards were used (note that equivalent cards could be used), they are:
  • NI PXI-5124 150 MHz, 200 MS/s, 12-Bit, 2 channel, 8 MB/channel, PXI Oscilloscope
  • 2X PXI-5421 43 MHz, 16-Bit, 1 Channel, PXI Waveform Generator
3. Insert the cards and plug a thunderbolt cable from the computer into the chassis card.
4. The last step is wiring all the parts together. This will be done mainly with SMA, SMB, and BNC cables. Refer to Fig. 17 All parts can be found in the bill of materials. Parts needed at this stage will be:
  • Assembled PCB’s
  • Magnet
  • 24 V DC power supply
  • RF power amplifier
  • Low-noise amplifier (LNA)
  • PXI Chassis with cards
  • 3X male SMA cables
  • 3X female SMB to male SMA cables
  • 2X power and ground to SMA connector
  • 2X BNC to SMA cables
  • 2X Male Pin SMA adapter (right angle)
5. To start connect male pin SMA adapters (right angle adapters) to both sides of the low noise amplifier then screw the *RF IN* side into the *LNA_IN* port and the *RF OUT* side into the *MIXER_IN* port.
6. Next solder a wire for both power and ground from the control board to their respective pins on the amplifier.
7. Wire the control board to the NI card using the SMA to BNC and SMA to SMB cables. Refer to Fig. 18.
8. Now that everything is connected correctly, obtain the magnet tray found in the Design files summary section. This tray is used to hold the coil PCB at the center of the magnet where the signal will be at its strongest.
9. Insert the coil PCB into the magnet tray, ensuring that a sample can easily slide into the coil. See Fig. 19.
10. Now slide the tray into the magnet.
11. Connect the impedance matching PCB to the port labeled *Impd_Match* on the control board.

**Fig. 20:**
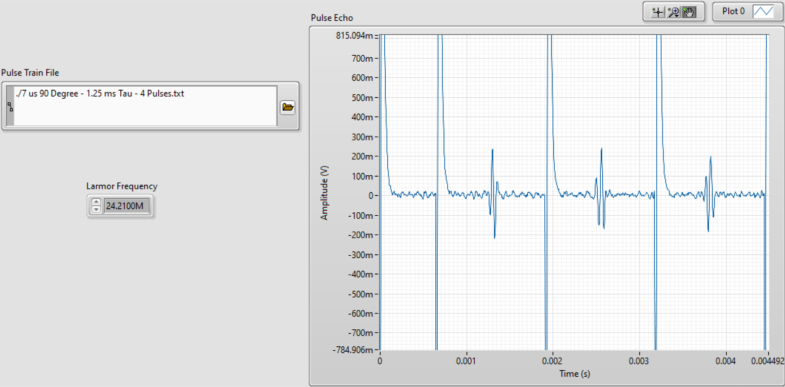
NMR single shot pulse sequence with a strong signal.

**Fig. 21:**
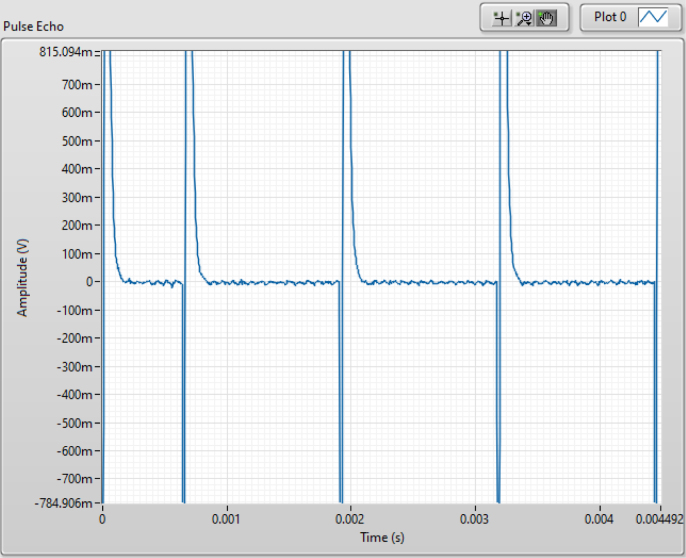
NMR single shot pulse sequence with no pulse resonance.

**Fig. 22:**
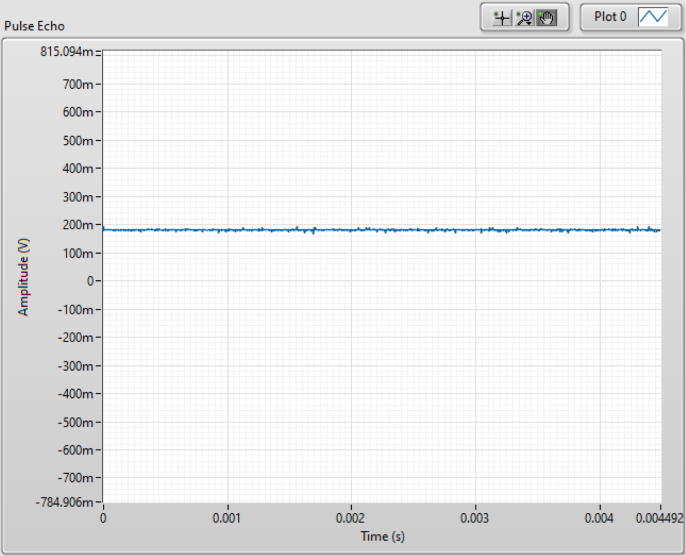
NMR single shot pulse sequence with no signal.

## 6 Operation instructions

This section outlines the operation instructions for the NMR system.

### 6.1 Software interface

All LabVIEW code is provided in the repository and its setup process is critical for operating the compact NMR system effectively, allowing users to control and monitor the performance of the NMR experiments. The setup involves configuring the software to interact with the hardware setup described in previous sections, specifically the PXI cards required to interact with the NMR pulse sequences. The detailed steps:

1. Begin by turning the PXI chassis on and turning on the control board’s power supply.
2. Open < NMR Single Shot Pulse Sequence.vi > in LabVIEW. The single shot pulse sequence is used for calibrating the system because you can quickly test for a signal.
3. Once open, in the front panel of LabVIEW enter the Larmor frequency for your system and open < 7us 90 Deg-1.25 ms Tau-4 Pulses.txt > in the pulse train file section.
4. Run the system. Your results will probably look like one of these:
  • If everything is perfect the results should look similar to those in Fig. 20. If this is the case you can move on to Section 6.2 and skip the following steps.
  • If the system is set up correctly but the Larmor frequency is not precise the results could look similar to those in Fig. 21. If this is the case adjust the Larmor frequency in LabVIEW by small intervals until you pick up a signal. It is best practice to use coarse adjustments of 0.01 MHz and fine adjustments of 0.001 MHz. Wait approximately 10 s between frequency adjustments to allow the nuclei to fully relax before testing again. If the signal is unobtainable re-calibration of the probe may be necessary.
  • If a component is not plugged in properly or lacks proper continuity in the system, you will likely see a flat line as shown in Fig. 22. Note that not only is there no signal, but also a DC offset. This will only happen if power is off or the system lacks proper continuity. To solve this problem, first, ensure that everything is correctly connected and that the power supply is turned on. An easy mistake that could cause improper connections is by putting two cords channels on the NI PXI-5124 or the NI PXI-5421 rather than one cord on a channel and one on a trigger. Refer to Fig. 18 for the proper connections. If the issue persists, proceed with these troubleshooting steps:
    – Check if the coil has been pushed up or down. If so, push it so that it lies inbetween the solder points
    – Check to be sure the tray is properly placed in the magnet
    – Check to be sure the sample in the tube is within the bounds of the coil
    – Check to be sure all SMA cables are tightened as needed
    – If the issue persists after these steps, it is likely that even though all wires are tightened, there is an incomplete connection somewhere. In this case, remove all wires and redo the wiring

### 6.2 Running a test

This section outlines the steps for conducting a test run using < NMR T2 CPMG Sequence.vi >. This program is designed to evaluate the functionality of the compact NMR system. Follow these instructions to configure the sequence parameters, execute multiple scans, and assess the system’s performance through visualized results.

**Fig. 23:**
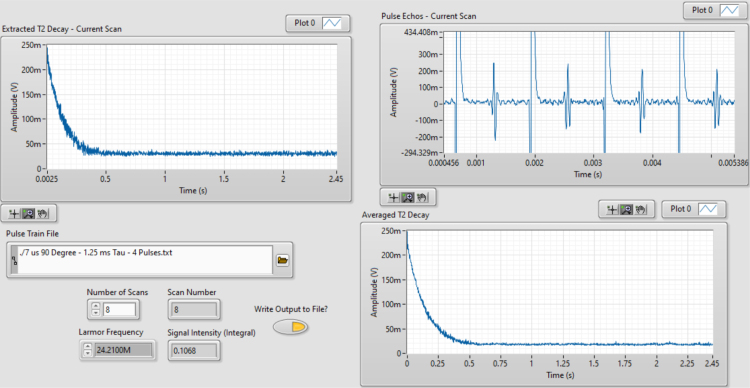
NMR $T_{2}$ CPMG Sequence results.

1. Open < NMR T2 CPMG Sequence > in LabVIEW.
2. In the front panel of LabVIEW enter the Larmor frequency for your system and open <7 μs 90 Degree - 0.7 ms Tau - 5000 Pulses.txt> in the pulse train file section.
3. Choose the number of scans you want the system to collect up to 8 (resolution increases with the number) and hit run.
4. If the system was properly calibrated and the single shot results were strong the results should look similar to Fig. 23:

Note that other CPMG pulse sequences may be useful for different applications. The pulse sequence files mentioned in this paper were generated using <pulse_sequence_creator_PXI5421.ipynb>. This Matlab program can be used to generate new pulse sequences for different pulse lengths and values of τ. The optimization of pulse sequences is left to the reader to allow for tuning of the pulse sequences to their measurement goals and hardware.

## 7 Validation and characterization

The accuracy of the compact TD-NMR system was tested in the copper $T_{2}$ relaxation study discussed here. Copper solutions were prepared by dissolving Copper Sulfate in deionized water resulting in concentrations ranging from 0 to 200 ppm of Copper(II) sulfate. These solutions were placed in 5 mm diameter test tubes so that the sample could be placed in the magnet tray and maintained at a centered position in the magnet. This is vital because the $T_{2}$ relaxation rate is dependent upon the Larmor frequency of the magnet. To maintain consistent data throughout measurements, the placement of the tube in the coil and magnet must remain constant, highlighting the importance of the magnet tray. Furthermore, since temperature also changes the Larmor frequency of the magnet, it is important to ensure that this remains constant throughout the measurement.

**Fig. 24:**
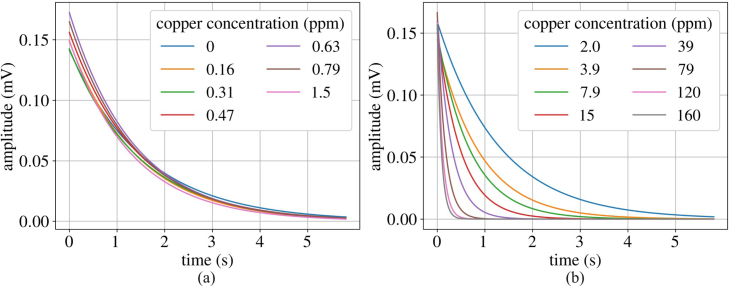
Fitted data showing the $T_{2}$ curves for varying concentrations of dissolved copper, showing: (a) ppm from 0 to 1.5 ppm and (b) ppm from 2 to 160 ppm.

Each measurement consisted of four NMR scans that detect pulse echos over a 3.5 s window. The maximum value of each spin echo is read and after the scan is complete, the maxima are plotted with respect to time, resulting in a $T_{2}$ curve. Each measurement takes the mean of the four $T_{2}$ curves from the scans, resulting in one set of data per measurement. Every concentration of copper was measured three times with this criteria. The $T_{2}$ time is defined as the time it takes for the magnetization of the spin echo to decay to 37 percent of its maximum value, according to:

(5) $$M_{xy}\left(t\right)=M_{0}\exp\left(-t/T_{2}\right),$$

where $M_{xy}\left(t\right)$ is the transverse magnetization at time t, $M_{0}$ is the magnetization at thermal equilibrium, and $T_{2}$ is the $T_{2}$ time. Using a non-linear least squares fit to the exponential equation, we acquired $T_{2}$ times and fitted $T_{2}$ curves for each of the samples. The fitted data for these curves can be seen in Fig. 24.

**Fig. 25:**
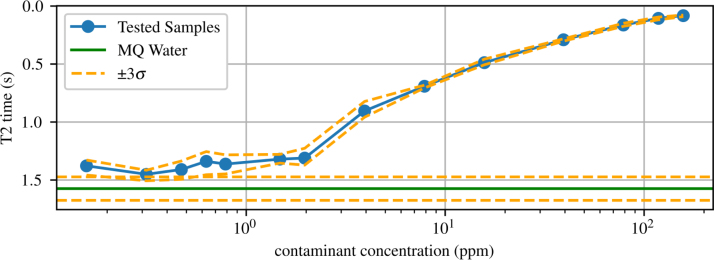
Copper $T_{2}$ time with varying concentration.

After collecting the $T_{2}$ times for each copper sample, the relationship between copper concentration and $T_{2}$ time was examined. It has been shown previously that increasing copper concentration in a fluid leads to a decrease in $T_{2}$ time [34]. Fig. 25 shows the relationship measured between $T_{2}$ time and copper concentration in ppm, highlighting an agreement with the previous study. Furthermore, within three standard deviations, the system is capable of measuring concentrations of copper dissolved in deionized water of around 900 ppb. The deionized water was purified using a Milli-Q water purification system (i.e. MQ water). This brief $T_{2}$ relaxation study showcases the TD-NMR system’s ability to measure pollutants in water systems by comparing the $T_{2}$ curves to those of water.

While this NMR system is capable of distinguishing copper-saturated water from deionized water at concentrations up to 900 ppb, there are some limitations to what can be measured. Since this is an (^1^H) NMR system, it can only detect molecules bonded with Hydrogen like fuels and oils as well as materials that affect Hydrogen magnetically, such as paramagnetic materials like copper ions. It is unable to detect ions like aluminum and zinc that are dissolved in water, unless something else is added that allows Hydrogen to bond with them. Some current limitations for the system include performance under temperature variability and the need to test deionized water as well to be a baseline for the measurement.

**Fig. 26:**
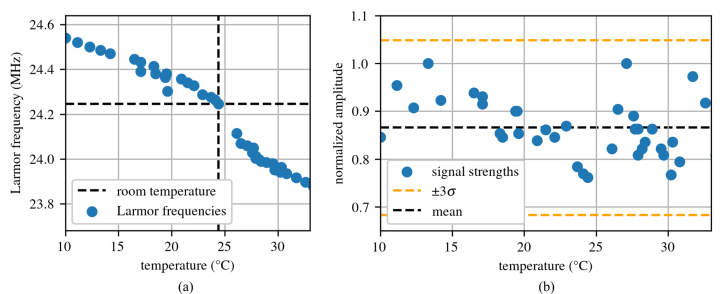
Temperature variance of the system showing: (a) how the Larmor frequency changes due to changes in the magnet’s temperature and (b) how the initial signal strength changes due to changes in the magnet’s temperature.

To test the temperature variability of the system, the magnet was cooled and heated to 10 and 33 °C respectively while both the amplitude of the signal and the Larmor frequency were measured. In this range, the signal strength of the magnet remains constant within error, but the Larmor frequency varies. Because of this, if the system were to be deployed in-situ, either temperature control, automated Larmor frequency tracking, or a combination of both would be required. Fig. 26 shows the results from this exploration.

This system is designed to be adaptable and scalable for many different purposes. For purposes demanding higher resolution, the coil can be adapted to a micro-coil. Usually increasing resolution requires increasing the homogeneity of the magnet, but an alternative is to use a smaller coil to decrease the inhomogeneity of the measuring region. For purposes involving in-situ data acquisition, the system can be temperature-controlled to avoid any loss of signal and necessary Larmor frequency tracking [24]. For remote data acquisition, the system can be adjusted using an NI PXI controller rather than a laptop communicating with the PXIe chassis via Thunderbolt. Furthermore, if the NI PXI systems present a financial challenge, then it is possible to utilize this hardware in a more inexpensive, but more difficult to implement, FPGA-based system [35]. There are many ways to optimize and adapt this system for specific needs.

## CRediT authorship contribution statement

**Winford Janvrin:** Writing – review & editing, Writing – original draft, Visualization, Validation, Software, Methodology, Investigation. **Jacob Martin:** Writing – original draft, Visualization, Validation, Software, Methodology, Investigation. **Daniel Hancock:** Writing – review & editing, Writing – original draft, Visualization, Validation, Methodology, Investigation, Formal analysis, Data curation. **Austin R.J. Downey:** Writing – review & editing, Writing – original draft, Visualization, Supervision, Project administration, Methodology, Funding acquisition, Conceptualization. **Perry J. Pellechia:** Methodology. **Joud Satme:** Methodology, Investigation. **Sang Hee Won:** Supervision, Funding acquisition, Conceptualization.

## Declaration of competing interest

The authors declare the following financial interests/personal relationships which may be considered as potential competing interests: Austin Downey reports financial support was provided by National Science Foundation. Austin Downey reports financial support was provided by Army Research Office. Authors declare that they have no known competing financial interests or personal relationships that could have appeared to influence the work reported in this paper.
